# *Rinorea niccolifera* (Violaceae), a new, nickel-hyperaccumulating species from Luzon Island, Philippines

**DOI:** 10.3897/phytokeys.37.7136

**Published:** 2014-05-09

**Authors:** Edwino S. Fernando, Marilyn O. Quimado, Augustine I. Doronila

**Affiliations:** 1 Department of Forest Biological Sciences, College of Forestry and Natural Resources, The University of the Philippines – Los Baños, College, 4031 Laguna, Philippines; 2 Analytical and Environmental Chemistry Research Group, School of Chemistry, University of Melbourne, Victoria 3010, Australia

**Keywords:** Hyperaccumulator, *Rinorea*, serpentine soils, ultramafics, Violaceae

## Abstract

A new, nickel-hyperaccumulating species of *Rinorea* (Violaceae), *Rinorea niccolifera* Fernando, from Luzon Island, Philippines, is described and illustrated. This species is most similar to the widespread *Rinorea bengalensis* by its fasciculate inflorescences and smooth subglobose fruits with 3 seeds, but it differs by its glabrous ovary with shorter style (5 mm long), the summit of the staminal tube sinuate to entire and the outer surface smooth, generally smaller leaves (3–8 cm long × 2–3 cm wide), and smaller fruits (0.6–0.8 cm diameter). *Rinorea niccolifera* accumulates to >18,000 µg g^-1^ of nickel in its leaf tissues and is thus regarded as a Ni hyperaccumulator.

## Introduction

*Rinorea* Aublet (Violaceae) is a pantropical genus of forest shrubs and trees. It is the second most species-rich genus in the family after *Viola* L., with an estimated total of 225–275 species throughout the tropics (Wahlert and Ballard 2012). In the Malesian region, only 11 species are recognized in the genus, with four species attributed to the Philippines (Jacobs and Moore 1971). However, the very broad circumscriptions of the widespread *Rinorea bengalensis* (Wall.) Kuntze and *Rinorea javanica* (Blume) Kuntze in the taxonomic revision of Jacobs and Moore (1971) needs a closer re-examination. A few new taxa from Borneo have subsequently been added for the region (e.g. Forman and Ahmad 1996, Jarvie and Stevens 1998, Stevens 2000).

In *Rinorea*, at least three species are known to hyperaccumulate the heavy metal nickel. *Rinorea bengalensis* (Wall.) Kuntze was the first nickel hyperaccumulator species of *Rinorea* discovered with up to 17,500 µg g^-1^ (dry weight) based on herbarium specimens from throughout Southeast Asia, including the Philippines (Brooks and Wither 1977). A subsequent analysis of herbarium material of 70 other species of *Rinorea* from Central and South America, Africa, and Asia also revealed another species, *Rinorea javanica* (Blume) Kuntze, as a nickel hyperaccumulator with up to 2,170 µg g^-1^ in its leaf tissues (Brooks et al. 1977). More recently, Proctor et al. (1994) reported another, yet unnamed, nickel-hyperaccumulating species of *Rinorea* from Mt Piapi on Karakelong Island, northeast of Sulawesi in Indonesia with up to 1,830 µg g^-1^ foliar Ni.

The Violaceae also includes two other genera that hyperaccumulate nickel. In *Agatea* A.Gray, one species from New Caledonia, *Agatea longipedicellata* (Baker f.) Guillaumin & Thorne, has been recorded to accumulate up to 2,500 µg g^-1^ of nickel in its foliar tissues (Jaffré 1980, Boyd and Jaffré 2009). In *Hybanthus* Jacq., at least six taxa from New Caledonia, Western Australia, and Sri Lanka are also known nickel hyperaccumulators (Reeves 2006) with maximum recorded foliar nickel levels of 1,860 µg g^-1^ in *Hybanthus enneaspermus* (L.) F.Muell. (Rajakaruna and Bohm 2002) to 25,500 µg g^-1^ in *Hybanthus austrocaledonicus* Melch. (Brooks 1994, Reeves 2003).

The ability to absorb certain metals and metalloids (chemical elements with properties in between those of metals and non-metals, also referred to as semi-metals) from the soil and to accumulate them in shoot tissues in exceptionally high and normally toxic concentrations without any evidence of physiological stress is rather rare among plants (Baker and Brooks 1989; Reeves and Baker 2000; Kramer 2010). Metal hyperaccumulation has recently been suggested to have had multiple origins within the angiosperms (Cappa and Pilon-Smits 2013). The more than 500 plant taxa thus far recorded as metal hyperaccumulators represent only a very small portion of all known angiosperms (Reeves and Baker 2000, Kramer 2010, van der Ent et al. 2012). The largest number of species, approximately 450, distributed in a wide range of angiosperm families, hyperaccumulate the metal nickel and generally occur on serpentine or ultramafic soils (van der Ent et al. 2012, Pollard et al. 2014).

Apart from their unusual and interesting ecology and physiology, hyperaccumulator plants have received considerable attention owing to the possibility of exploiting their accumulation traits for practical applications, especially in the development of so-called environmentally green technologies, e.g. phytoextraction, phytoremediation of heavy metal in contaminated soils, or phytomining to recover commercially valuable metals in plant shoots from mineralized sites (Chaney et al. 1997, Brooks and Robinson 1998, McGrath and Zhao 2003, Reeves 2003, Pilon-Smits 2005, Rascio and Navari-Izzo 2011).

In the Philippines, much of the forest flora on ultramafic or serpentine soils (Fernando et al. 2008) remain underexplored. Field surveys in a number of sites in the archipelago have revealed some new species (e.g. Hoffmann et al. 2003, Fernando and Rodda 2013), including several species that are able to accumulate heavy metals in their above-ground tissues (Baker et al. 1992, Hoffmann et al. 2003, Fernando et al. 2013, Gotera et al. 2014). In this paper, we describe a new species of *Rinorea* discovered in remnant forest on ultramafic soils that is also a nickel-hyperaccumulator. This species is, thus far, known only from small populations in the northern section of Zambales Province on Luzon Island in the Philippines. This area is part of the Zambales Ophiolite Complex (Rossman et al. 1989, Zhou et al. 2000, Yumul 2004) which is host to several metallic mineral deposits (e.g. chromium, nickel) (Osberger et al. 1988, Bacuta et al. 1990, Yumul et al. 2003).

## Materials and methods

The morphology of the species presented here was based on field, vegetative, and reproductive characters. Field characters were recorded on site. Vegetative characters were observed and measured from press-dried specimens and seedlings and reproductive characters from fresh specimens and from material preserved in 70% ethanol. Detailed morphological measurements were made using digital calipers and a calibrated eye piece under a dissecting microscope. Herbarium specimens were also consulted and compared at CAHUP, LBC, PNH, and PUH, including additional material, e.g. images of type specimens of Southeast Asian and Philippine *Rinorea* available online at BISH, K, L, MO, NY, and US. All photographs, except where indicated, were taken in the field in the natural habitat of the species. Conservation threat assessment follows IUCN Categories and Criteria (IUCN 2012).

Field semi-quantitative screening for nickel accumulation in this species was performed on site on leaf samples, thoroughly washed in distilled water, crushed in a mortar and pestle, and tested on filter paper previously impregnated with 1% of the nickel-specific colorimetric reagent, dimethylglyoxime, dissolved in 95% ethanol (Baker et al. 1992; Reeves et al. 1996, 1999). Formation of pink or magenta color indicated exceptionally high (above 1,000 µg g^-1^) concentration of Ni in the dry plant matter. Tissue samples of roots, stems and leaves, and of soil from the rhizosphere (*c.* 30–100 cm) of each plant sampled were also collected. These were subsequently subjected to laboratory elemental analyses for nickel (Ni) and two other heavy metals, copper (Cu) and cobalt (Co). The plant samples were thoroughly washed in distilled water and then oven-dried at 60 °C. Each sample was later weighed into borosilicate test tubes and ashed in a muffle furnace for 4–5 hours, with the final temperature of 500 °C being maintained for the last 2 hours. The ash was then taken up in 5 ml of warm 2 M HCl and the digest finally made up to an appropriate volume (5–20 ml) then analyzed for Ni, Cu, and Co content using atomic absorption spectrophotometer. The soil samples were digested with aqua regia (3:1 concentrated hydrochloric acid: nitric acid), then diluted appropriately for metal analyses of Ni, Cu, and Co using an atomic absorption spectrophotometer. Details of this method follow in general that described by Reeves et al. (1996).

## Results and discussion

### Taxonomy

#### Rinorea niccolifera

Fernando sp. nov.

urn:lsid:ipni.org:names:77138469-1

http://species-id.net/wiki/Rinorea_niccolifera

Figures 1 , 2

##### Diagnosis.

*Rinorea niccolifera* is most similar to *Rinorea bengalensis* by its fasciculate inflorescences and smooth subglobose fruits with 3 seeds, but it differs by its glabrous ovary with shorter style (5 mm long), the summit of the staminal tube sinuate to entire and the outer surface smooth, and its generally smaller leaves (3–8 cm long × 2–3 cm wide) and smaller fruits (0.6–0.8 cm diameter).

TYPE: PHILIPPINES. Luzon Island: Zambales Province, Municipality of Sta. Cruz, Lucapon, in remnant forest on ultramafic soils, along a gully with large boulders, 330 m elevation, flowers and immature fruits, 01 April 2012, *Fernando 3016* (holotype LBC; isotypes CAHUP, K, PNH, SING).

##### Description.

*Shrub* or small *tree*, 1.5–8 m tall; stem 3–13 cm diameter, outer bark generally smooth, inner bark whitish; young twigs rather zigzag, with prominent stipular scars. *Leaves* simple, distichous, lamina elliptic to narrowly obovate, (2–) 3–8 (–10) cm long × (1–) 2–3 (–4) cm wide; the margins finely serrate, especially towards the distal half; base acute; apex acute to acuminate; secondary nerves (6–) 8–12 (–13) on each side of the midrib, diverging 40–60° from the midrib; hairy pit domatia very prominent along the midrib on abaxial surface; petiole terete, (–2) 3–5 (–7) mm long; young leaves white or greenish-white, growing in flushes. *Stipules* narrowly lanceolate, (4–) 6–7 (–8) mm long × 1 mm wide at the base, prominently covering the apical bud, caducous and leaving a distinct scar. *Flowers* white or cream, bisexual, globose or broadly ovoid, 3.1–3.3 mm long × 3.1–4 mm wide, in dense axillary clusters or fascicles of up to 3–5, sometimes more, rarely solitary; pedicel 2.5–3.2 mm long, 0.7–0.9 mm wide, sparsely covered with fine, short hairs. *Sepals* 5, free, subequal in size and shape, nearly as wide as long, broadly ovate, 1.3–1.6 × 1.3–1.6 mm, shorter than the petals, light green or greenish-white, distinctly 2–4 (–5) veined, margins entire, ciliate towards the distal half and sometimes covered with brown fine hairy indumentum at the apex. *Petals* 5, free, subequal in size and shape, broadly oblong to ovate, the apex rounded or obtuse, 2.2–2.7 mm long × 1.3–1.7 mm wide, white or greenish-white, paler towards the apex, the tip slightly deflexed or recurved, margins smooth or sometimes slightly ciliolate near the apex. *Stamens* 5; anther with 2 thecae, 1 mm long × 0.6 mm wide; connective appendage broadly ovate, 0.4 mm long × 0.7 mm wide, membranous, cream or light orange, the margins fimbriate; filaments as long as the tube, 0.6 mm long × 0.2 mm wide, inserted on the inner surface of the staminal tube surrounding the ovary; staminal tube shallowly 5-lobed, 0.6 mm tall and 0.7 mm thick, the summit sinuate to entire, outer and inner surfaces glabrous, smooth. *Ovary* ovoid, glabrous, smooth, 1 mm long, 0.9–1 mm diameter, with 3 locules; ovule 1 each per locule; style 0.5 mm long × 0.3 mm wide, erect; stigma pointed, undifferentiated. *Fruit* a capsule, globose or depressed globose, obscurely 3-angular, 6.5–7 mm long × 6–8 mm wide, green, turning pale green when ripe, glabrous, subtended by the persistent sepals and petals; remnant of stigma prominent, 1–1.5 mm long; 3-locular, dehiscent along three sutures, the locules 7–8 mm long, 5 mm wide and 4 mm deep, folding inwards when seeds are released; pedicel 4–5 mm long, 1–1.5 mm thick. *Seeds* 3, one in each locule, globose, 3–4 mm long × 3 mm wide, mottled light brown; hilum distinct, white. *Seedling* with epigeal germination, phanerocotylar; cotyledons foliaceous, 8 mm long × 10 mm wide, apex slightly emarginate, base truncate or obtuse; eophylls simple, elliptic, spirally arranged, 11 mm × 5 mm, margins serrate.

**Figure 1.**
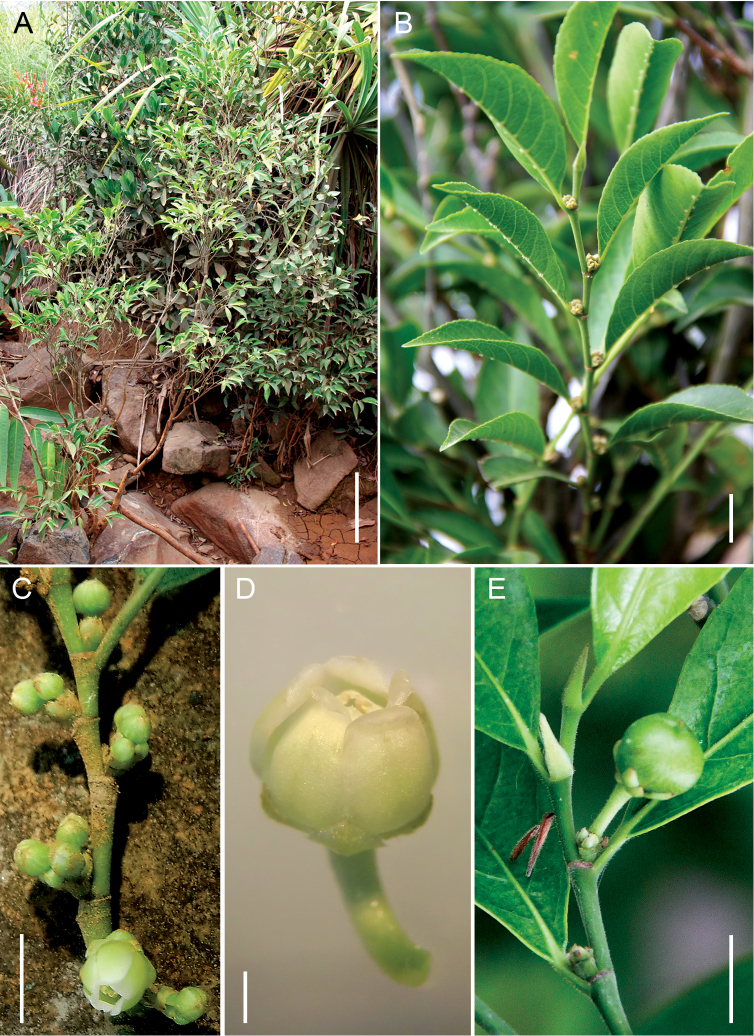
*Rinorea niccolifera* Fernando. **A** Growth habit at type locality **B** Twig with flower buds and leaves showing distinct domatia on abaxial surface **C** Twig with flowers in axillary sessile clusters or fascicles, some on young, leafless portions of the twig; note prominent stipular scars **D** Close-up of flower, showing recurved tips of petals **E** Twig with young fruit subtended by the persistent sepals and petals; note the caducous stipules. Scale bars: **A** = 20 cm; **B, E** = 10 mm; **C** = 5 mm; **D** = 1 mm **A**–**E** from *Fernando 3016* (LBC). All photographs by Edwino S. Fernando.

**Figure 2.**
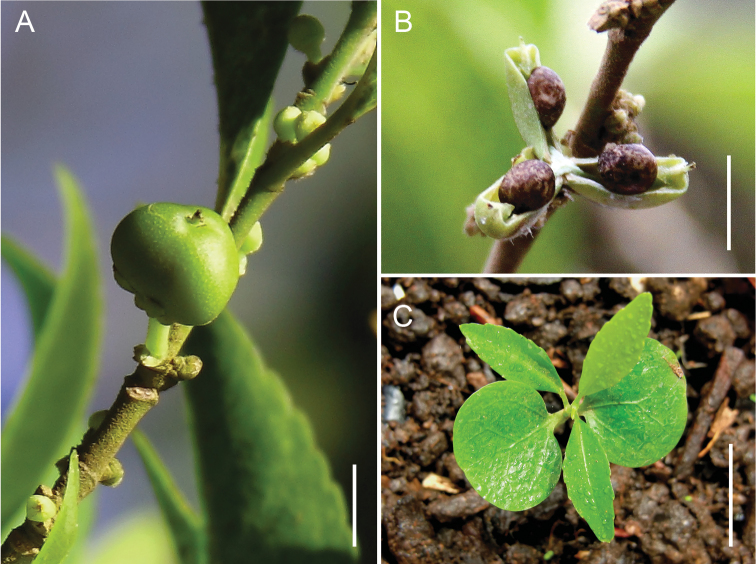
*Rinorea niccolifera* Fernando. **A** Immature fruit showing depressed globose or obscurely 3-angular shape with prominent stylar remains **B** Mature, dehisced fruit showing three locules and seeds **C** Seedling showing foliaceus cotyledons and first three eophylls. Scale bars: **A, B** = 5 mm; **C** = 10 mm. **A** from *Fernando 3016* (LBC), **B** from *Fernando 3042* (LBC), **C** from *Fernando 3042A* (LBC), from seed of *Fernando 3042* germinated in nursery. All photographs by Edwino S. Fernando.

##### Distribution.

Endemic in the Philippines. Luzon Island, Zambales Province, Municipalities of Sta. Cruz and Candelaria.

##### Habitat and ecology.

This species grows in forests on ultramafic soils, usually along gullies or sloping areas with large boulders or rocks at elevations of 320–825 m. In its type locality, *Rinorea niccolifera* was observed growing with *Syzygium longissimum* (Merr.) Merr. (Myrtaceae), *Clerodendrum klemmei* Elmer (Lamiaceae), *Ixora ebracteolata* Merr. (Rubiaceae), *Severinia disticha* (Blanco) Swingle (Rutaceae), *Diospyros ferrea* (Willd.) Bakh. (Ebenaceae), *Calophyllum pentapetalum* (Blanco) Merr. (Calophyllaceae), *Dillenia luzoniensis* (Vidal) Merr. (Dilleniaceae) and *Terminalia pellucida* C.Presl (Combretaceae), among several other woody plant species. In some other sites within its range in the ultramafic area of Sta. Cruz and Candelaria in northern Zambales, *Rinorea niccolifera* may occur together with *Rinorea bengalensis*, the latter also a nickel hyperaccumulator, but is generally a larger tree reaching to 15 m tall and with stem diameter of up to 25 cm (see also further in Key to the species).

##### Additional specimens examined.

Philippines, Luzon Island, Zambales Province, Municipality of Sta. Cruz, Lucapon, along a gully with large boulders, 320 m elevation, flower buds, 19 April 2011, *Fernando 2421* (K, LBC, PNH), *Fernando 2422* (LBC, PNH); flower buds, 01 April 2012, *Fernando 3015* (CAHUP, LBC, PNH), mature fruits and seeds, 26 May 2012, *Fernando 3042* (LBC, PNH); Municipality of Candelaria, Malimlim area, on steep slope with rocky soil, 630 m elevation, sterile material, 18 January 2013, *Fernando 3072* (LBC), juvenile flower buds, 18 January 2013, *Fernando 3073* (LBC, PNH), 605 m elevation, sterile material, 18 May 2013, *Fernando 3161* (CAHUP, LBC), on steep slope, 750 m elevation, sterile material, 18 May 2013, *Fernando 3181* (LBC), on ridge summit, 825 m elevation, sterile material, 19 November 2013, *Fernando 3338* (LBC, PNH); Cultivated: Laguna Province, Los Baños, seedlings grown from seeds of *Fernando 3042* germinated in nursery, 21 August 2012, *Fernando 3042A* (LBC).

##### Etymology.

The specific epithet *niccolifera* refers to the ability of this species to hyperaccumulate the heavy metal nickel in its stem and leaf tissues (from *niccolum* – Neo Latin for nickel, and; *fer* – to yield, to contain).

##### Conservation status.

Following the IUCN Categories and Criteria (IUCN 2012), we regard this species as Endangered (EN B2ab(ii,iii,iv)). Its habitat is severely fragmented and is so far recorded only from three adjacent localities. Its current known area of occupancy is estimated to be less than 500 km^2^, and a continuing decline is observed, inferred or projected in its (a) extent of occurrence; (b) area of occupancy; and (c) area, extent and/or quality of habitat. Much of the habitat of this new species is subject to open pit mining.

### Key to the species of *Rinorea* in the Philippines

**Table:** 

1	Ovules 6; lateral nerves often 16 or more on either side of the midrib	*Rinorea horneri*
–	Ovules 3; lateral nerves often less than 16 on either side of the midrib	2
2	Inflorescences more or less elongate, 0.5–2.6 cm long	*Rinorea javanica*
–	Inflorescences fasciculate, or flowers densely set on short rachis <0.5 cm long	3
3	Fruit ovoid, sparsely hairy; leaves without domatia on abaxial surface	*Rinorea macrophylla*
–	Fruit subglobose, smooth; leaves with domatia on abaxial surface	4
4	Ovary hairy; style 1 mm long; outer surface of staminal tube puncticulate, the summit irregularly lobed; fruit broader, 1–1.5 cm diameter; leaves generally larger, (6–) 9–16 (–22) cm long × (3–) 4–9 (–10) cm wide; stipules 9–13 (–14) mm long	*Rinorea bengalensis*
–	Ovary glabrous; style 0.5 mm long; outer surface of staminal tube smooth, the summit slightly sinuate to entire; fruit narrower, 0.6–0.8 mm diameter; leaves generally smaller, (2–) 3–8 (–10) cm long × (1–) 2–3 (–4) cm wide; stipules often less than 8 mm long	*Rinorea niccolifera*

### Metal hyperaccumulation in *Rinorea niccolifera*

**Figure 3.**
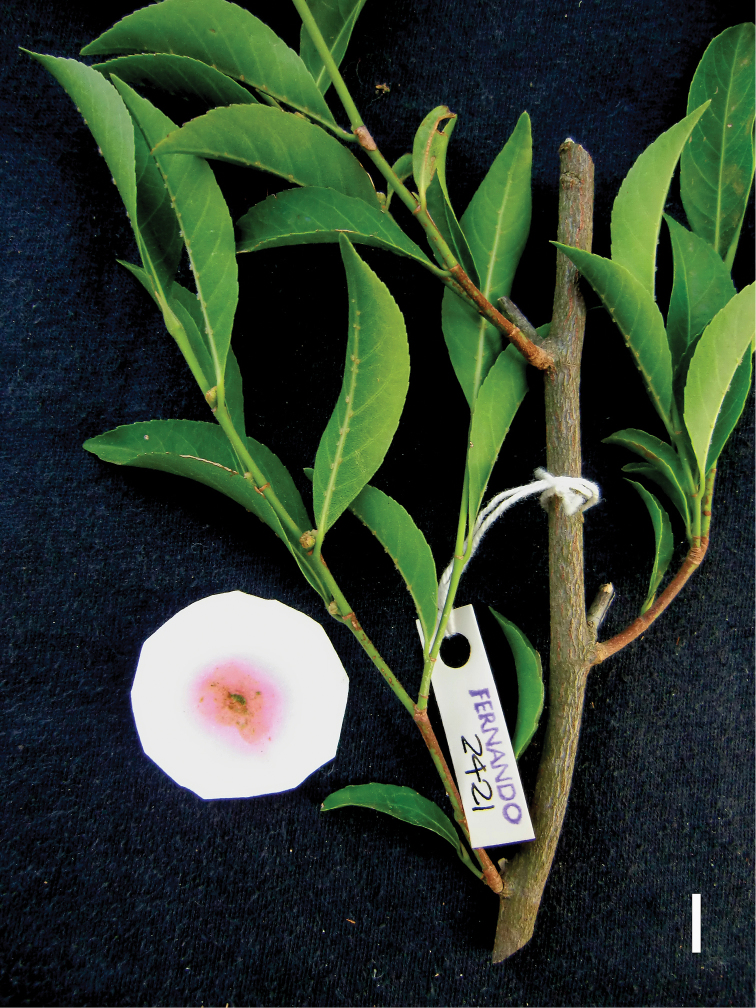
*Rinorea niccolifera* Fernando, shown as a nickel hyperaccumulator by a field test using filter paper impregnated with 1% dimethylglyoxime dissolved in 95% ethanol. Scale bar = 10 mm. *Fernando 2421* (LBC). Photograph by Edwino S. Fernando.

Field screening for Ni accumulation in *Rinorea niccolifera* using the colorimetric reagent, dimethylglyoxime (Baker et al. 1992, Reeves et al. 1996, 1999) indicated high levels in the leaves (Figure 3). Subsequent chemical analyses of the plant tissues in the laboratory revealed foliar nickel concentrations varying from 7,168 to 18,388 µg g^-1^ on dry weight basis (Table 1). The data shown in Table 1 is based on six sets of plant tissue samples of *Rinorea niccolifera* collected from two sites. The range of foliar Ni concentration on dry weight basis is similar to that reported for *Rinorea bengalensis* with 15,400–17,500 µg g^-1^ (Brooks and Wither 1977, Reeves 2003, Jopony and Tongkul 2011) on ultramafic soils. It is, however, higher when compared with *Rinorea javanica*, 2,170 µg g^-1^ (Brooks et al. 1977) or *Rinorea* sp., 1,830 µg g^-1^ (Proctor et al. 1994). As this species surpasses the 10,000 µg g^-1^ Ni accumulation level in the leaves, it is regarded as a ‘hypernickelophore’ following the Ni accumulation category of Jaffré and Schmid (1974) and Boyd and Jaffré (2009). The cobalt (Co) accumulation in *Rinorea niccolifera* (21.35–51.46 µg g^-1^) (Table 1) was low but is within the range recorded by Brooks et al. (1977) for *Rinorea bengalensis* (0.5–545 µg g^-1^) and *Rinorea javanica* (3–670 µg g^-1^). All these figures are above the normal concentrations (0.03–2 µg g^-1^) of cobalt in plants, which according to Reeves (2006) rarely exceeds 20 µg g^-1^. Copper (Cu) accumulation (Table 1) was also within normal range of concentrations (5–25 µg g^-1^) for plants (Reeves 2006).

**Table 1.** Mean and range values (in brackets) of Ni, Cu, and Co concentrations in leaves, stems, roots and rhizospheric soil of *Rinorea niccolifera* at two sites in Zambales Province, Luzon Island, Philippines. All concentrations are in µg g^-1^ dry matter. Mean values shown with standard errors.

Site	Nickel (Ni)	Copper (Cu)	Cobalt (Co)
**Sta. Cruz**			
Leaves^1^	13334.17 ± 1872.63	6.99 ± 0.14	37.66 ± 4.28
	(7168.27 – 17986.43)	(<5.00 – 7.37)	(27.56 – 51.46)
Stems	1880.51 ± 765.49	9.53^3^	5.0 ± 1.21
	(779.34 – 4147.55)	(<5.00 – 9.53)	(<2.50 – 6.21)
Roots	1036.93 ± 163.22	7.19 ± 1.42	4.26^3^
	(592.72 – 1331.18)	(<5.00 – 8.62)	(<2.50 – 4.26)
Soil^2^	3981.54 ± 747.89	78.66 ± 11.64	579.75 ± 19.51
	(1869.54 – 5042.54)	(45.18 – 94.5)	(546.27 – 611.24)
**Candelaria**			
Leaves^1^	17497.69 ± 890.67	<5.00	23.69 ± 2.34
	(16607.01 – 18388.36)	<5.00	(21.35 – 26.03)
Stems	4742.94 ± 1964.77	7.44 ± 0.55	3.73 ± 0.06
	(2778.16 – 6707.71)	(6.89 – 8.00)	(3.66 – 3.79)
Roots	3060.93 ± 307.11	<5.00	3.27 ± 0.13
	(2753.82 – 3368.04)	<5.00	(3.14 – 3.40)
Soil^2^	2756.04 ± 1021.89	241.36^3^	363.36 ± 56.41
	(1734.15 – 3777.94)	(<5.00 – 241.36)	(306.96 – 419.77)

^1^ Voucher specimens for materials used for chemical analyses are as follows: Sta Cruz, 4 samples – *Fernando 2421, 2422, 3015, 3016*; Candelaria, 2 samples – *Fernando 3073, 3161*.

^2^ Total metal concentration in rhizospheric soil.

^3^ Based on single specimen record; others in the sample were below the set detection levels of 5.00 µg g^-1^ for Cu and 2.50 µg g^-1^ for Co.

## Supplementary Material

XML Treatment for Rinorea niccolifera
